# Human umbilical cord mesenchymal stromal cells as an adjunct therapy with therapeutic hypothermia in a piglet model of perinatal asphyxia

**DOI:** 10.1016/j.jcyt.2020.10.005

**Published:** 2021-06

**Authors:** Nicola J. Robertson, Christopher Meehan, Kathryn A. Martinello, Adnan Avdic-Belltheus, Tiziana Boggini, Tatenda Mutshiya, Ingran Lingam, Qin Yang, Magdalena Sokolska, Xenia Charalambous, Alan Bainbridge, Mariya Hristova, Boris W. Kramer, Xavier Golay, Ben Weil, Mark W. Lowdell

**Affiliations:** 1 Institute for Women's Health, University College London, London, UK; 2 University College London Hospitals NHS Foundation Trust, London, UK; 3 Department of Pediatrics, University of Maastricht, Maastricht, the Netherlands; 4 Royal Free London NHS Foundation Trust, London, UK

## Abstract

**Background:** With therapeutic hypothermia (HT) for neonatal encephalopathy, disability rates are reduced, but not all babies benefit. Pre-clinical rodent studies suggest mesenchymal stromal cells (MSCs) augment HT protection. **Aims:**The authors studied the efficacy of intravenous (IV) or intranasal (IN) human umbilical cord-derived MSCs (huMSCs) as adjunct therapy to HT in a piglet model. **Methods:**A total of 17 newborn piglets underwent transient cerebral hypoxia-ischemia (HI) and were then randomized to (i) HT at 33.5C 113 h after HI (n=7), (ii) HT+IV huMSCs (30נ10^6^ cells) at 24 h and 48 h after HI (n=5) or (iii) HT+IN huMSCs (30נ10^6^ cells) at 24 h and 48 h after HI (n=5). Phosphorus-31 and hydrogen-1 magnetic resonance spectroscopy (MRS) was performed at 30 h and 72 h and terminal deoxynucleotidyl transferase dUTP nick end labeling (TUNEL)-positive cells and oligodendrocytes quantified. In two further piglets, 30נ10^6^ IN PKH-labeled huMSCs were administered. **Results:**HI severity was similar between groups. Amplitude-integrated electroencephalogram (aEEG) recovery was more rapid for HT+IN huMSCs compared with HT from 25 h to 42 h and 49 h to 54 h (*P* 0.05). MRS phosphocreatine/inorganic phosphate was higher on day 2 in HT+IN huMSCs than HT (*P*=0.035). Comparing HT+IN huMSCs with HT and HT+IV huMSCs, there were increased OLIG2 counts in hippocampus (*P*=0.011 and 0.018, respectively), internal capsule (*P*=0.013 and 0.037, respectively) and periventricular white matter (*P*=0.15 for IN versus IV huMSCs). Reduced TUNEL-positive cells were seen in internal capsule with HT+IN huMSCs versus HT (*P*=0.05). PKH-labeled huMSCs were detected in the brain 12 h after IN administration. **Conclusions:**After global HI, compared with HT alone, the authors saw beneficial effects of HT+IN huMSCs administered at 24 h and 48 h (30נ10^6^ cells/kg total dose) based on more rapid aEEG recovery, improved ^31^P MRS brain energy metabolism and increased oligodendrocyte survival at 72 h.

## Introduction

Neonatal encephalopathy (NE) is an important cause of death and disability, affecting one to three out of 1000 births in the UK [1] and 1025 out of 1000 births in Sub-Saharan Africa [2]. With the current practice of therapeutic hypothermia (HT), NE mortality has reduced from 25% to 9% and disability from 20% to around 16%, with a reduction in the rate of cerebral palsy [3]. However, not all children benefit from treatment, and intellectual impairment may remain even in the absence of cerebral palsy [4].

Over the last 10 years, several agents have been studied as adjunct therapies for HT in babies, but there is currently no clinically recommended therapy, apart from HT, for NE [5]. The use of stem cells to successfully treat neonatal brain injury is emerging as a promising therapy, with mesenchymal stromal cells (MSCs) as the lead product under investigation [6]. MSCs are attractive because of their low immunogenicity (allowing non-matched allogeneic transplantation), multilineage differentiation and secretome with pluripotent effects of cytokines, chemokines, growth factors and extracellular vesicles, reacting to the needs of the ischemic cerebral environment [7]. Human umbilical cord-derived mesenchymal stromal cells (huMSCs) have a high differentiation potential and superior anti-inflammatory properties compared with MSCs from adult tissues [8]. This, combined with the developmentally active phase of the newborn brain, leads to a high efficiency of huMSCs. Importantly, after transplantation, MSCs home to the areas of injury [9]. They do not survive long term or replace damaged tissues themselves but react to the needs of the environment by secreting growth factors and cytokines to regulate damage and repair, auto-tuning to the brain's milieu [10]. These intrinsic adaptive properties of huMSCs make them excellent candidates to treat the devastating effects of NE. Indeed, stem cell pilot studies have been completed for NE (autologous umbilical cord cells administered intravenously, NCT00593242), and clinical trials are underway for perinatal arterial ischemic stroke (50 million allogeneic bone marrow-derived MSCs administered intranasally, NCT03356821) and bronchopulmonary dysplasia (30 million huMSCs/kg administered intratracheally, NCT03558334) [7]. Safe and feasible MSC doses used in babies are ~20נ10^6^ cells/kg, which is comparable to animal models [9].

The aims of the study were to (i) assess whether two doses of 30 million huMSCs at 24 h and 48 h augment hypothermic neuroprotection (~2-kg piglet, total treatment dose 30נ10^6^ huMSCs/kg), (ii) compare neuroprotective efficacy of intravenous (IV) versus intranasal administration of 30 million huMSCs at 24 h and 48 h and (iii) assess whether PKH26-labeled huMSCs migrate to the brain tissue by detection of PKH26 immunofluorescence 12 h after intranasal administration. Primary outcome measures were (i) cerebral magnetic resonance spectroscopy (MRS) biomarkers (phosphorus-31 [^31^P] and hydrogen-1 [^1^H])^31^P MRS phosphocreatine/inorganic (PCr/Pi) ratio defines secondary energy failure in NE [11] and is associated with 1-year brain growth and outcome [12], and ^1^H MRS thalamic lactate/N-acetylaspartate (Lac/NAA) predicts outcome at 2 years [13,14] and is used in clinical neuroprotection trials [15], with clear superiority over other magnetic resonance (MR) methods [13]; (ii) amplitude-integrated electroencephalogram (aEEG) recoveryaEEG is used in NE babies during HT and rate of recovery of electrical activity predicts neurodevelopmental outcome [16]; and (iii) quantitative cell death (terminal deoxynucleotidyl transferase dUTP nick end labeling [TUNEL]-positive cells) and oligodendrocyte survival in eight brain regions at 72 h.

## Methods

### Animal experiments, surgical preparation and randomization

All animal experiments were approved by the University College London Ethics Committee and performed according to UK Home Office Guidelines (Animals [Scientific Procedures] Act, 1986). The study followed Animal Research: Reporting of *In Vivo* Experiments guidelines. The model has been modified since its first description by Lorek *et al.* [17] in 1994. The authors recent modifications include a longer experimental duration of 72 h rather than 48 h; continuous aEEG/EEG monitoring throughout the study, except during magnetic resonance imaging (MRI); cerebral hypoxia-ischemia titrated to aEEG and mean arterial blood pressure (MABP) rather than ^31^P MRS; use of an MRI-compatible incubator for intensive care; and transfer to a clinical 3T MR system twice during the study. In brief, newborn large, white male piglets aged <48 h were sedated with intramuscular midazolam (0.2 mg/kg) and anesthetized with isoflurane mixed with air (3% v/v during surgery, 1.52.5% during experimentation), remaining insentient throughout experimentation. Animals were mechanically ventilated via tracheostomy (SLE 2000 infant ventilator; Surrey, UK) and settings guided by arterial blood gas analysis (Pao_2_, 813 kPa, Paco_2_, 4.56.5 kPa). The common carotid arteries were surgically isolated and carefully encircled by inflatable carotid occluders (OC2A; In Vivo Metric). An umbilical arterial line was inserted for MABP and heart rate monitoring and umbilical venous line for infusions. Infusions included maintenance 10% dextrose at 60 mL/kg/day (reduced to 40 mL/kg/day post-insult), fentanyl 36 mcg/kg/h and antibiotics (benzylpenicillin 50 mg/kg/dose 12 hourly, gentamicin 5 mg/kg/dose once daily). The arterial line was infused with heparinized saline (0.5 IU/mL in 0.9% sodium chloride) at 0.3 mL/h.

Animals were nursed prone in a purpose-built MR-compatible transport incubator. Temperature was maintained at 38.5C during normothermia and at 33.5C during the 12-h period of HT with a servo-controlled cooling blanket placed around the piglet in the pod (Tecotherm; Inspiration Healthcare, Crawley, UK). Rewarming was performed over 10 h at 0.5C/h. Intensive care was provided throughout the 72-h experiment, and complications (e.g., hypotension, seizures, hyperkalemia) were treated as per local neonatal guidelines. To maintain MABP >40mmHg, 0.9% saline bolus (10 mL/kg), dopamine (520 g/kg/min), dobutamine (520 g/kg/min), noradrenaline (0.11.5 g/kg/min) and adrenaline (0.11.5 g/kg/min) were used as required by a neonatologist.

**Fig. 1:**
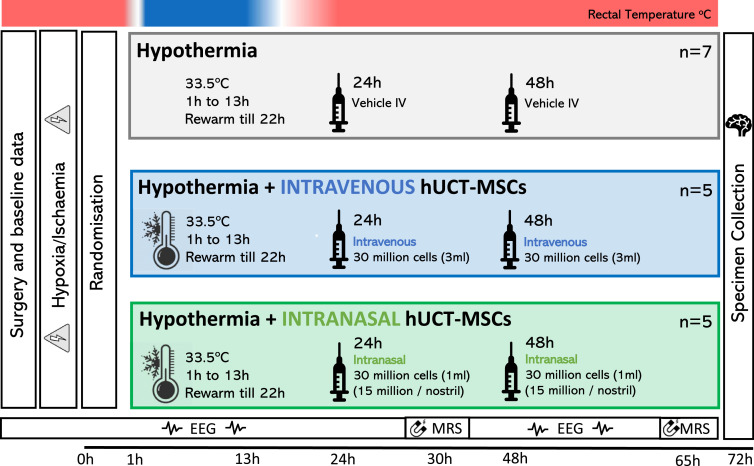
Study timeline. Following baseline data acquisition, piglets underwent global cerebral HI. At the end of HI (time 0), piglets were randomized to (i) HT at 33.5C 113 h after HI, with saline bolus at 24 h and 48 h (n=7); (ii) HT+IV huMSCs (30 million cells in 3.0 mL ) at 24 h and 48 h after HI (n=5); or (iii) HT+IN huMSCs (30 million cells in 1.0 mL ) at 24 h and 48 h after HI (n=5). Intensive care was given for 72 h following HI, and aEEG/continuous video EEG was acquired. MRI/MRS was performed at 3T at 30 h and 65 h. The experiment was terminated at 72 h and brain immunohistochemistry analyzed. (Color version of figure is available online).

A six-channel EEG (Nicolet) using clinical-grade needle electrodes was set up and EEG data acquired at baseline, during hypoxia-ischemia (HI) and throughout the experiment (removed only during MRI scans). Both the raw EEG and aEEG trace were evaluated throughout the study. The criteria for study entry were (i) normal aEEG/EEG at baseline after surgery, (ii) no pyrexia and (iii) no aEEG recovery within 1 h of HI. The experimental plan is shown in Figure 1.

### Transient cerebral HI

Baseline physiological observations and aEEG were monitored prior to HI. Cerebral HI was commenced by simultaneously inflating the carotid occluders remotely and reducing the fraction of inspired oxygen (Fio_2_) at the start of insult as previously described [18]. Fio_2_ was decreased to 6% over the first 3 min and titrated to MABP and EEG. Oxygen delivery was increased in the event of a mean MABP <27 mmHg and restricted further if recovery of EEG activity was observed during the insult. Blood gas analysis was performed at 5-min intervals during HI. Total duration of HI was optimized to be 2025 min, depending on the duration of isoelectric EEG, hypotension (mean blood pressure <30 and <25 mmHg), total reduction in Fio_2_ (area under the curve Fio_2_) and severity of acidosis on blood gas analysis. At the end of the insult, the animal was resuscitated, occluders deflated and Fio_2_ increased to air.

Following HI and resuscitation, animals were randomized to (i) therapeutic hypothermia from 1 h to 13 h (HT) plus IV 0.9% saline (3 mL) at 24 h and 48 h, (ii) HT plus 30נ10^6^ IV huMSCs (HT+IV huMSCs) at 24 h and 48 h (3 mL IV) or (iii) HT plus 30נ10^6^ intranasal (IN) huMSCs (HT+IN huMSCs) at 24 h and 48 h (0.5 mL in each nostril). Intensive care support for the animal throughout 72 h and complications were managed in accordance with local neonatal intensive care guidelines.

### The huMSC preparation

MSCs were derived from a single umbilical cord donor tissue supplied by the Anthony Nolan Cord Blood Bank and Cell Therapy Centre under Human Tissue Authority license. The tissue was manually dissected, then enzymatically digested to isolate MSCs using plastic adherence. Passage zero cells were cultured in serum-containing Minimum Essential Medium Eagle-Alpha Modification (Gibco) with antibiotic/antimycotic at 5% CO_2_ at 37C. When confluent, the flasks were harvested with TrypLE Select (Gibco), then expanded in serum- and xeno-free cell culture medium (Biological Industries) to form a cell stock at passage two. Subsequent cell expansion utilized closed-system, single-use bioreactors (Xpansion, Pall Corporation) until the product was harvested at passage four with a maximum of 30 population doublings. A 64-fold increase (i.e., six population doublings) was achieved during bioreactor expansion. MSCs were tested for purity (>95% CD73, CD105, CD90 and <2% CD34, CD45, CD14, CD19 and HLA-DR) and sterility (absence of aerobic, anaerobic bacteria and fungi). Although the MSCs were from a single umbilical cord donor, analysis of >20 clinical umbilical cord donors as well as pooled donor products and genetically modified products suggested donor consistency in terms of growth kinetics (data not shown), ability to suppress T-cell proliferation (see supplementary file) and phenotypic characteristics (following International Society for Cell & Gene Therapy criteria) when expanded to >40 population doublings.

#### The huMSC phenotyping

MSC immunophenotyping was assessed by flow cytometry analysis using NovoCyte (ACEA Biosciences), conducted by trained operators (Table 1). Cells were stained with 1 L of each antibody for 20 min in the dark at room temperature before washing and resuspending for acquisition. Cells were gated on forward scatter versus side scatter, doublets excluded by plotting height against area, then negative/positive cutoffs determined against a fluorescence-minus-one control. An antibody cocktail of anti-human CD90-PE (clone 5E10), CD73-PE-Vio770 (clone AD2), CD105-APC (clone 43A4E1), CD14-FITC (clone M5E2), CD19-FITC (clone HIB19), CD34-FITC (clone 581), CD45-FITC (clone HI30) and HLA-DR (clone TU36) was used for immunophenotyping.

**Table 1:** Flow cytometry panel for MSC immunophenotyping.

Fluorochrome	Anti-human antibody	Manufacturer/supplier	Cat. No.
FITC	CD14	BD Biosciences	561712
	CD19	BD Biosciences	555412
	CD34	BD Biosciences	560942
	CD45	BD Biosciences	560976
	HLA-DR	BD Biosciences	555560
PE	CD90	BD Biosciences	561970
PE-Vio770	CD73	Miltenyi Biotec	130-104-225
APC	CD105	Miltenyi Biotec	130-094-926

APC, allophycocyanin; Cat., category; FITC, fluorescein isothiocyanate; PE, phycoerythrin.

#### MSC PKH labeling

The huMSCs were stained with the lipophilic dye PKH26 (Sigma-Aldrich,Gillingham,UK)according to the manufacturer's protocol prior to cryopreservation.Briefly, cells were incubated in Diluent C and PKH26 for 15 min at room temperature. The reaction was halted with human albumin solution and then the cell suspension centrifuged at 400 g for 10 min and resuspended in fresh medium. The washing procedure were repeated three additional times before a cell count was performed (using Trypan Blue exclusion), followed by formulation and cryopreservation of MSCs.

#### Formulation and cryopreservation

MSCs were formulated in either 90% human albumin solution (Zenalb 4.5; Bio Products Laboratory) and 10% dimethyl sulfoxide (DMSO) (Wak-Chemie) at 15נ10^6^ cells in 1.5 mL per cryovial (for IV administration) or PRIME-XV MSC FreezIS DMSO-free solution (Irvine Scientific) at 15נ10^6^ cells in 0.5 mL per cryovial (for IN administration). All cells were frozen passively using a Mr. Frosty in a 80 freezer and then transferred to vapor-phase nitrogen for long-term storage. The cell number administered was calculated pre-freezing. The authors validated the recovery and viability post-thaw as previously described [19] and observed a viability of >80% in both DMSO and non-DMSO cryopreserved cells. Cells were administered directly after thawing and without washing or re-culture.

### Administration

#### IV administration

In the IV huMSC group, two cryovials (2נ15נ10^6^ huMSCs/1.5 mL) were defrosted at room temperature before an immediate slow bolus into the cephalic indwelling catheter at 24 h and 48 h after HI. A total volume of 3.0 mL containing 30נ10^6^ cells was given IV at 24 h and 48 h.

#### IN administration

In the IN huMSC group, two cryovials (each 15נ10^6^ huMSCs/0.5 mL) were defrosted at room temperature. The animal was elevated by 45 degrees and the head further tilted upward. One vial of IN huMSCs was administered to each nostril using a neonatal nasogastric tube, cut to size and inserted to the back of the nasal passage, the position of which was previously established using MRI. Cells were injected slowly into the IN space adjacent to the olfactory bulbs. The authors did not use hyaluronidase to disrupt the nasal epithelium and facilitate cell penetration.

In two further animals, following transient HI, PKH26-labeled huMSCs were administered intranasally, and the experiment was terminated at 12 h. One hemisphere was cryopreserved and the other processed in the same way as the whole brain in the other 17 animals (paraformaldelhyde and paraffin embedding), as described later.

### Magnetic resonance spectroscopy

The ^1^H and ^31^P MR spectra were acquired at 24 h and 48 h after HI on a Philips clinical 3T MRI scanner. The ^31^P MRS metabolites were acquired using a 6-cm diameter circular transmit receive coil (PulseTeq, Chobham, UK) placed above the piglet's head. A single-pulse acquisition with an average repetition time of 10 seconds and 32 seconds was used.

The ^1^H MRS metabolites were acquired using chemical shift imaging, performed with a repetition time of 2 seconds and a TE of 288 ms. Voxels were 8נ8נ10 mm^3^ over an 8נ8 matrix. The spectral width was 2 kHz with 2048 points. Data from the voxels over the left basal ganglia and thalamus and the left subcortical white matter (WM) at the level of the centrum semiovale were selected and processed using the open-source Tarquin software (http://tarquin.sourceforge.net/) by a physicist blinded to the treatment group. The Lac/NAA peak area ratio represents lactate+threonine/total N-acetylaspartate+N-acetylaspartylglutamate, as the inclusion of threonine and total NAA in the spectral fitting has been shown to optimize accurate prediction of neurodevelopmental outcome in babies with NE [20].

### Amplitude-integrated electroencephalogram

Multichannel EEG and aEEG (Nicolet) were acquired at baseline and continued for 72 h post-insult. The aEEG was scored by two assessors blinded to the treatment allocation, and the score was based on pattern classification [21]: isoelectric (0), continuous low voltage (1), burst suppression (2), discontinuous normal voltage (3) and continuous normal voltage (4).

### Immunohistochemistry

Piglets were euthanized using pentobarbital (1 g/kg) either 72 h after HI with unlabeled huMSCs (n=17) or 12 h after HI with PKH26-labeled huMSCs (n=2). Organs were fixed through a transcardial perfusion with cold phosphate-buffered saline, followed by 4% phosphate-buffered paraformaldehyde. The whole brain (n=17) was dissected out and post fixed at 4C in 4% paraformaldehyde for 7 days. Coronal slices (5-mm thick) were embedded in paraffin wax and sectioned (5 m). For each animal, two sections, one taken through the hippocampus (R1) and another 5 mm anterior (R0), were assessed for each stain.

For PKH26 huMSCs (623 and 624, n=2), the left hemisphere was removed prior to paraformaldehyde perfusion and dissected into 0.5-cm coronal slices. Slices were coated in optimal cutting temperature mounting medium and rapidly frozen in isopentane chilled to 150C with liquid nitrogen and stored at 80C. To detect the PKH26-labeled huMSCs, coronal frozen sections were counterstainedwithProLong gold antifade mountant with 4,6-diamidino-2-phenylindole (DAPI)(Invitrogen, Paisley, UK) beforefluorescentmicroscopy wasperformedusing an Olympus IX70 inverted fluorescence microscope (Olympus Europa, Hamburg, Germany)andMicropixCytocam 2.0.3.0 software.

All paraffin-embedded brain sections were dehydrated in xylene (3נ10 min) and rehydrated in graded ethanol solutions (10070%), followed by double-distilled water. For TUNEL, the sections were pre-treated for 15 min in 3% hydrogen peroxide in methanol to remove endogenous peroxidase, followed by a 15-min peptidase pre-digestion with 20 g/mL proteinase K (Promega) at 65C, and then incubated at 37C for 2 h with the TUNEL solution (Roche) containing biotinylated dUTP. For CC3, Iba1 and OLIG2, pre-treatment was performed with Ventana CC1 (950-124), equivalent to ethylenediaminetetraacetic acid buffer, for 32 min. For GFAP, protease 1 (0.38 mg/mL alkaline protease enzyme activity) was used for 4 min. Incubation with primary rabbit antibody was performed against CC3 (1:100) (9661L; Cell Signaling Technology) for 32 min, Iba1 (1:250) (019-19741; Wako) for 4 h, GFAP (1:1000) (Z0334; Dako) for 32 min and OLIG2 (1:100) (AB9610; Millipore) for 4 h. Incubation with a secondary swine anti-rabbit immunoglobulin (E0343; Dako) was performed for 44 min (CC3), 1 h (Iba1 and OLIG2) or 32 min (GFAP).

Biotin residues were detected with the avidin-biotinylated horseradish peroxidase complex (Vector Laboratories) and visualized with diaminobenzidine/hydrogen peroxide (Sigma-Aldrich), with cobalt chloride and nickel chloride included to intensify TUNEL histochemistry. The sections were dehydrated in graded alcohol and xylene and mounted with Depex (VWR) or, alternatively, Vectashield plus DAPI aqueous mounting media (Vector Laboratories) to facilitate total cell number counts during analysis of Iba1 and CC3.

Investigators blind to the treatment group performed analyses in eight brain regions (cingulate cortex, sensorimotor cortex, hippocampus, periventricular white matter, internal capsule, caudate nucleus, putamen and thalamus). For each section and brain region, TUNEL+ nuclei were counted in three fields (at 40 magnification, with an area of 0.066 mm^2^) and the average converted into counts per mm^2^. Iba1-positive cell body count was similarly performed. In addition, Iba1-positive microglial cell bodies and branch density were calculated at 40 magnification using a 0.049נ0.049-mm 15-square grid placed in three fields for all brain regions, counting the number of cell bodies within the grid (defined as value C) and the average number of branches crossing the three horizontal and three vertical 0.49-mm gridlines (defined as value B). The microglial ramification index was calculated as B^2^/C. CC3 immunoreactive cells were counted in three fields (at 20 magnification, with an area of 0.164 mm^2^) and the average converted into counts per mm^2^. To quantify GFAP immunoreactivity, optical luminosity values were calculated by deducting mean brightness values of the tissue (three fields per region at 20 magnification) from the mean brightness of the blank region of the corresponding slide [22]. Optical luminosity value is an established technique used for assessment of glial cells, taking into account the fact that most of the time not just cell bodies but also processes are stained [22].

### Statistical analysis

Statistical analysis was performed using Prism 6.0 for Mac (GraphPad Software, La Jolla, CA, USA). Parametric data were analyzed using Student's *t*-test and non-parametric data with Mann-Whitney U test. MRS, aEEG and immunohistochemistry data were analyzed using analysis of variance of the least mean squared difference. Graphical methods were used to assess the distribution of the results to assess the appropriate statistical approach. If histology data were positively skewed and not normally distributed, data were log_10_ transformed to normalize distribution for parametric statistical analysis [23].

## Results

Nineteen piglets were studied overall. Seventeen piglets were included in the efficacy study: HT (n=7), HT+IV huMSCs (n=5) and HT+IN huMSCs (n=5). Two piglets were studied with PKH-labeled huMSCs for cell tracking at 12 h after HI.

### Physiological data and insult severity

There were no intergroup differences for body weight, baseline heart rate, MABP or core temperature**.** Arterial blood gases at baseline were similar. Considering HI severity, the duration of (i) hypoxia and carotid occlusion, (ii) isoelectric EEG and (iii) MABP <25 and <30 mmHg was similar. At the end of HI, blood pH, lactate, base excess and area under the curve Fio_2_ reduction below 21% were similar (Table 2). There was no difference in inotrope use or saline boluses needed to maintain MABP within the normal range following HI (Table 3).

**Table 2:** Physiological parameters for piglets in each group.

Parameter	HT Least square mean (SEM)	HT+IV huMSCs Mean (SEM)	HT+IN huMSCs Mean (SEM)	*P* value
Weight (kg)	1.99 (0.03)	1.98 (0.04)	1.96 (0.04)	0.790
Hypoxic-ischemic insult				
Duration of HI (min)	23.57 (1.25)	23.40 (1.48)	23.00 (1.48)	0.957
Duration of isoelectric EEG during HI (min)	21.00 (1.21)	19.20 (1.43)	20.60 (1.43)	0.628
Duration of MABP <30 mmHg (min)	11.57 (1.260	7.6 (1.49)	7.6 (1.49)	0.088
Duration of MABP <25 mmHg (min)	2.00 (0.87)	0.8 (1.03)	2.60 (1.03)	0.468
Nadir pH	7.20 (0.03)	7.25 (0.04)	7.28 (0.04)	0.353
Nadir lactate (mmol/L)	12.48 (0.69)	12.77 (0.82)	11.48 (0.82)	0.513
Nadir base excess (mmol/L)	9.86 (1.48)	10.20 (1.75)	6.20 (1.75)	0.223
Area under the curve Fio_2_	299.71 (24.17)	327.40 (28.60)	308.60 (28.60)	0.763
Heart rate (min^1^)				
Baseline	176.4 (6.81)	170.2 (8.06)	158.0 (8.06)	0.250
01 h after insult	202.0 (8.18)	192.3 (9.67)	180.2 (9.67)	0.261
125 h	181.9 (11.49)	182.3 (13.59)	177.5 (13.59)	0.960
2549 h	193.6 (9.46)	182.7 (11.19)	174.9 (11.19)	0.451
4972 h	165.9 (6.78)	161.4 (8.02)	160.8 (8.02)	0.863
MABP (mmHg)				
Baseline	55.0 (1.91)	55.1 (2.25)	50.4 (2.25)	0.256
01 h after insult	53.7 (3.17)	51.4 (3.75)	44.1 (3.75)	0.177
125 h	44.1 (1.56)	44.5 (1.84)	45.8 (1.84)	0.781
2549 h	52.5 (1.85)	50.7 (2.18)	50.4 (2.18)	0.726
4972 h	51.2 (1.72)	54.9 (2.03)	50.9 (2.03)	0.322
Rectal temperature (C)				
Baseline	38.4 (0.17)	38.1 (0.20)	37.8 (0.20)	0.100
01 h after insult	38.2 (0.09)	38.1 (0.10)	38.0 (0.10)	0.310
125 h	35.0 (0.03)	34.9 (0.03)	35.0 (0.03)	0.916
2549 h	38.2 (0.13)	38.0 (0.16)	36.4 (0.1)	0.561
4972 h	38.0 (0.09)	38.0 (0.08)	38.4 (0.08)	0.138
Pao_2_ (kPa)				
Baseline	7.1 (0.51)	5.2 (0.61)	5.7 (0.61)	0.067
End of HI (time 0)	6.2 (0.54)	4.8 (0.63)	5.8 (0.63)	0.278
12 h	5.9 (0.63)	5.5 (0.75)	6.1 (0.75)	0.812
24 h	5.8 (0.44)	5.7 (0.52)	6.0 (0.52)	0.911
48 h	6.0 (0.29)	5.1 (0.35)	5.9 (0.35)	0.190
72 h	4.8 (0.25)	4.8 (0.29)	5.2 (0.29)	0.579
Paco_2_ (kPa)				
Baseline	14.2 (2.52)	15.2 (2.98)	18.1 (2.98)	0.614
End of HI (time 0)	3.7 (0.49)	4.9 (0.58)	3.8 (0.58)	0.271
12 h	11.5 (1.45)	10.3 (1.72)	13.3 (1.72)	0.477
24 h	13.3 (1.24)	12.8 (1.47)	14.6 (1.47)	0.661
48 h	14.5 (0.78)	12.8 (0.92)	13.6 (0.92)	0.387
72h	13.6 (1.29)	11.7 (1.52)	16.2 (1.52)	0.149
Blood pH				
Baseline	7.4 (0.04)	7.5 (0.04)	7.5 (0.04)	0.189
End of HI (time 0)	7.2 (0.03)	7.2 (0.04)	7.3 (0.04)	0.344
12 h	7.5 (0.03)	7.5 (0.04)	7.5 (0.04)	0.966
24 h	7.4 (0.03)	7.5 (0.04)	7.5 (0.04)	0.577
48 h	7.4 (0.02)	7.5 (0.03)	7.5 (0.03)	0.439
72 h	7.5 (0.03)	7.5 (0.03)	7.5 (0.03)	0.924
Base excess (mmol/L)				
Baseline	8.6 (1.83)	6.2 (2.17)	8.6 (2.17)	0.662
End of HI (time 0)	9.9 (1.48)	10.2 (1.75)	6.2 (1.75)	0.223
12 h	10.0 (1.35)	7.2 (1.59)	11.4 (1.59)	0.199
24 h	3.7 (2.41)	6.2 (2.86)	8.2 (2.86)	0.497
48 h	5.0 (1.51)	5.0 (1.78)	5.2 (1.78)	0.996
72h	3.6 (1.83)	3.8 (2.16)	6.2 (2.16)	0.624
Lactate (mmol/L)				
Baseline	2.2 (0.50)	3.6 (0.59)	3.0 (0.59)	0.211
End of HI (time 0)	12.5 (0.69)	12.8 (0.82)	11.5 (0.82)	0.514
12 h	3.3 (0.6)	3.9 (0.6)	3.1 (0.6)	0.487
24 h	3.3 (0.66)	3.7 (0.78)	2.3 (0.78)	0.128
48 h	**4.9 (0.88)**	**1.9 (1.05)**	**3.0 (1.05)**	**0.042**
72h	1.4 (0.20)	1.0 (0.24)	1.9 (0.24)	0.061
Glucose (mmol/L)				
Baseline	6.3 (0.84)	6.9 (1.00)	5.8 (1.00)	0.766
End of HI (time 0)	9.2 (1.31)	9.1 (1.55)	9.5 (1.55)	0.976
12 h	13.3 (1.84)	12.3 (2.18)	10.4 (2.18)	0.618
24 h	16.0 (3.01)	8.0 (3.56)	12.0 (3.56)	0.261
48 h	5.7 (0.48)	5.7 (0.63)	6.3 (0.57)	0.669
72h	7.3 (0.64)	5.5 (0.84)	6.0 (0.76)	0.216
Potassium (mmol/L)				
Baseline	4.3 (0.21)	4.4 (0.28)	4.6 (0.25)	0.577
End of HI (time 0)	4.5 (0.71)	5.0 (0.84)	3.9 (0.84)	0.641
12 h	6.1 (0.41)	5.8 (0.49)	6.0 (0.49)	0.254
24 h	6.8 (0.46)	6.1 (0.55)	6.6 (0.55)	0.307
48 h	6.9 (0.68)	4.5 (0.81)	6.4 (0.81)	0.657
72h	6.6 (0.67)	3.8 (0.79)	5.4 (0.79)	0.056

Time 0=time of resuscitation after HI. Least square mean values (SEM) are shown for the three groups: HT (n=7), HT+IV huMSCs (n=5) and HT+IN huMSCs (n=5). ANOVA model was fitted to each group at each time point and a Bonferroni multiplicity correction made. Bold figures represent those measurements that are significantly different between groups.

ANOVA, analysis of variance; SEM, standard error of the mean.

**Table 3:** Inotrope and saline bolus volume replacement for piglets in HT, HT+IV huMSC and HT+IN huMSC groups.

Infusions	HT Mean SD		HT+IV huMSCs Mean SD		HT+IN huMSCs Mean SD		*P* value
Dopamine (g/kg/min)	8.7	7.6	8.4	5.0	10.9	7.7	0.822
Dobutamine (g/kg/min)	1.6	2.1	2.7	2.5	1.5	2.7	0.692
Noradrenaline (ng/kg/min)	6.5	15.3	2.4	4.7	4.6	10.3	0.842
Adrenaline (ng/kg/min)	1.5	3.9	0.1	0.3	0.0	0.0	0.550
Saline bolus (10 mL/kg bolus)	0.0	0.0	0.2	0.4	0.2	0.4	0.504

SD, standard deviation.

### Amplitude-integrated EEG recovery and seizures

**Fig. 2:**
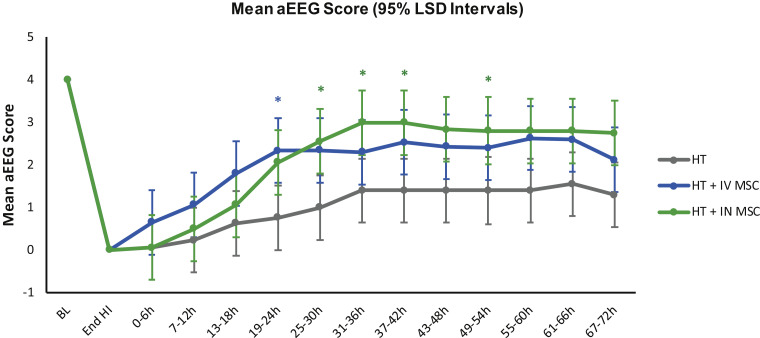
The aEEG background activity at baseline, during HI and following HI. The grouped mean hourly aEEG scores per 6-h period with 95% LSD are shown. All piglets had a normal aEEG background voltage (score 4) at the start of the experiment and a flat trace (score 0) in the first hour after HI insult. The mean hourly aEEG background voltage recovery was faster in the HT+IN huMSC group (n=5) compared with the HT group (n=7) from 25 h to 30 h, significance indicated by green* (*P*=0.043), 31 h to 36 h (*P*=0.039), 37 h to 42 h (*P*=0.039) and 49 h to 54 h (*P*=0.036). At 1924 h (before IV huMSC administration), the HT+IV huMSC group (n=5) had a higher aEEG score than the HT group (n=7), significance indicated by blue*, but after this point there was no difference between the HT+IV huMSC and HT groups. LSD, least significant difference. (Color version of figure is available online).

All piglets had a normal aEEG background voltage (score 4) at the start of the experiment and a flat trace (score 0) in the first hour after the HI insult. At 1924 h (before huMSC administration), the HT+IV huMSC group had a higher aEEG score than the HT group, but after this point there was no difference between the two groups (Figure 2).

Seizures were detected in five (35%) piglets. After HI, the first seizure was noted at 17 h and 22 h in the HT+IN huMSC group (before the first treatment), at 19 h in the HT+IV huMSC group (before the first treatment) and at 34 h and 37 h in the HT group. There was no difference between groups in numbers of piglets with seizures (*P*=0.69). All electrographic seizures were treated with 20 mg/kg phenobarbitone; short clinical seizures with no electrographic evidence of seizures were not treated.

### 3T MRS

**Fig. 3:**
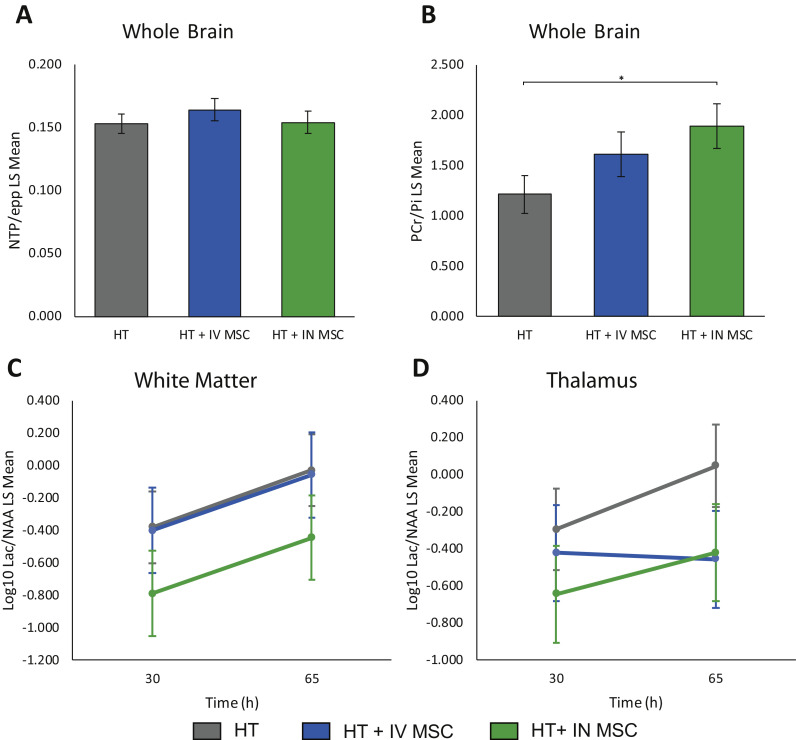
**Magnetic Resonance Spectroscopy.**The ^31^P MRS NTP/epp (A) and PCr/Pi (B) of the brain at 30 h after HI are shown in the top row. Comparing HT+IN huMSCs (n=5) with HT (n=7), there was higher PCr/Pi (*P*=0.035) (3B). There was no difference in NTP/epp between groups. The ^1^H MRS white matter (C) and thalamus (D) at 30 h and 65 h are shown in the lower row. In comparing HT+IN or IV huMSCs with HT, there was no difference between groups in thalamic or WM Lac/NAA. LS, least square; NTP/epp, nucleotide triphosphate/exchangeable phosphate pool. The * in B denotes statistcal significance between HT and HT+IN MSC. (Color version of figure is available online).

Comparing HT+IN huMSCs with HT, there was higher PCr/Pi (*P*=0.035) on day 2 (Figure 3). There was no difference in the nucleotide triphosphate/exchangeable phosphate pool between groups. Comparing HT+IN or IV huMSCs with HT, there was no difference between groups in basal ganglia and thalamus or WM Lac/NAA.

### Immunohistochemistry

#### Terminal deoxynucleotidyl transferase dUTP nick end labeling

**Fig. 4:**
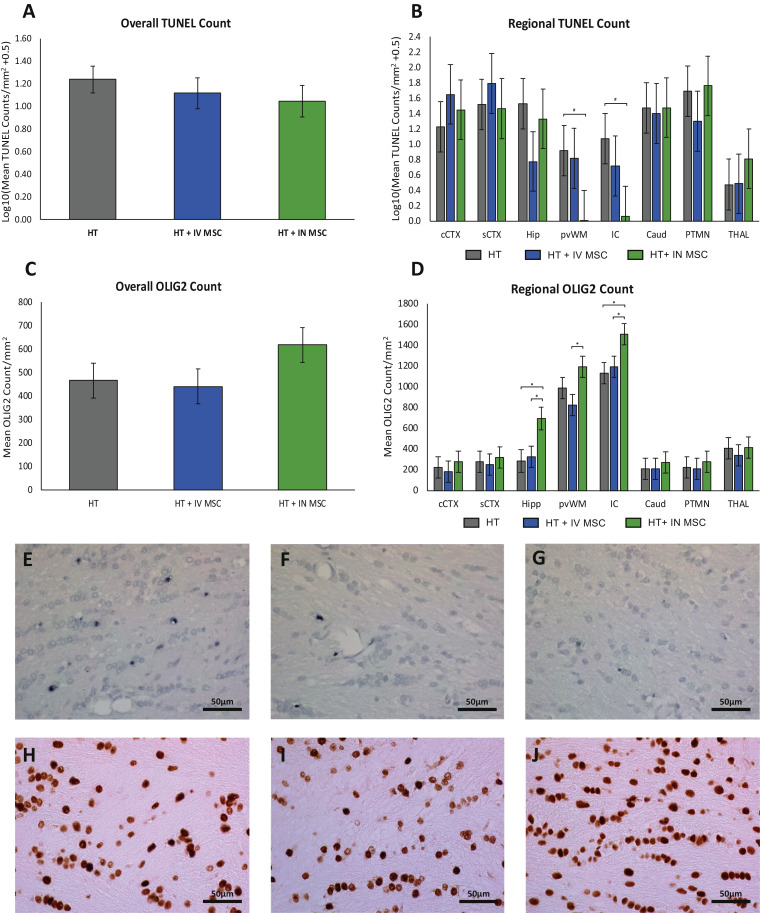
**Immunohistochemistry results for TUNEL and OLIG2.** TUNEL immunohistochemistry in (A) over all brain regions and (B) in eight individual brain regions are shown. Overall, there were no differences between groups. With regard to regional differences, there were fewer TUNEL-positive cells in the IC in the HT+IN huMSC group (n=5) versus the HT group (n=70). P =0.05 (IC) and P= 0.078 (pvWM). Representative TUNEL photomicrographs from the IC of HT (E), HT+IV huMSC (F) and HT+IN huMSC (G) groups are shown. (C) Mean OLIG2 cells/mm^2^ overall and (D) Mean OLIG2 cells/mm^2^ in eight individual brain regions are shown. There were higher OLIG2 counts in IC in the HT+IN huMSC group (n=5) versus the HT (n=7) (*P*=0.013) and HT+IV huMSC (n=5) (*P*=0.037) groups. Higher OLIG2 counts were observed in the hippocampus in the HT+IN huMSC group versus the HT (*P* < 0.011) and HT+IV huMSC (*P*=0.018) groups. In the pvWM, there were more OLIG2 cells in the HT+IN huMSC group compared with the HT+IV huMSC group (*P*=0.018). Representative OLIG2 photomicrographs from the IC of the HT (H), HT+IV huMSC (I) and HT+IN huMSC (J) groups are shown. *P < 0.05. Caud, caudate; cCTX, cingulate cortex; Hip, hippocampus; IC, internal capsule; PTMN, putamen; pvWM, periventricular white matter; sCTX, sensorimotor cortex; THAL, thalamus. (Color version of figure is available online).

TUNEL-positive cells/mm^2^ for treatment groups are shown in Figure 4, with group comparison in Table 4. Over all regions, TUNEL-positive cells/mm^2^ (unlogged) were 17.3 cells/mm^2^ in HT, 13.1 cells/mm^2^ in HT+IV huMSC and 11.1 cells/mm^2^ in HT+IN huMSC groups. Overall, there were no differences between groups. With regard to regional differences, there were lower TUNEL-positive cells in the internal capsule in the HT+IN huMSC versus HT group (*P*=0.050).

**Table 4:** Log_10_ TUNEL-positive cell counts/mm^3^.

Brain regions	HT	HT+IV huMSCs	HT+IN huMSCs	*P* values for differences in means	
cCTX	1.23 (0.33)	1.65 (0.39)	1.45 (0.39)	HT vs HT+IV huMSCs	0.406
				HT vs HT+IN huMSCs	0.662
				HT+IV huMSCs vs HT+IN huMSCs	0.715
sCTX	1.52 (0.33)	1.79 (0.39)	1.46 (0.39)	HT vs HT+IV huMSCs	0.589
				HT vs HT+IN huMSCs	0.919
				HT+IV huMSCs vs HT+IN huMSCs	0.552
Hippocampus	1.53 (0.33)	0.78 (0.39)	1.33 (0.39)	HT vs HT+IV huMSCs	0.143
				HT vs HT+IN huMSCs	0.701
				HT+IV huMSCs vs HT+IN huMSCs	0.315
pvWM	0.92 (0.33)	0.82 (0.39)	0.01 (0.39)	HT vs HT+IV huMSCs	0.847
				HT vs HT+IN huMSCs	0.078
				HT+IV huMSCs vs HT+IN huMSCs	0.145
IC	1.07 (0.33)	0.82 (0.39)	0.06 (0.39)	HT vs HT+IV huMSCs	0.491
				**HT vs** **HT+IN huMSCs**	**0.050**
				HT+IV huMSCs vs HT+IN huMSCs	0.236
Caudate	1.48 (0.33)	1.40 (0.39)	1.48 (0.39)	HT vs HT+IV huMSCs	0.880
				HT vs HT+IN huMSCs	0.998
				HT+IV huMSCs vs HT+IN huMSCs	0.888
Putamen	1.70 (0.33)	1.30 (0.39)	1.76 (0.39)	HT vs HT+IV huMSCs	0.442
				HT vs HT+IN huMSCs	0.897
				HT+IV huMSCs vs HT+IN huMSCs	0.406
Thalamus	0.48 (0.33)	0.49 (0.39)	0.81 (0.39)	HT vs HT+IV huMSCs	0.983
				HT vs HT+IN huMSCs	0.510
				HT+IV huMSCs vs HT+IN huMSCs	0.554
Overall	1.24 (0.12)	1.12 (0.14)	1.05 (0.14)	HT vs HT+IV huMSCs	0.506
				HT vs HT+IN huMSCs	0.289
				HT+IV huMSCs vs HT+IN huMSCs	0.714

cCTX, cingulate cortex; IC, internal capsule; pvWM, periventricular white matter; sCTX, sensorimotor cortex; SEM, standard error of the mean. Bold values denote statistical signfiicance.

#### OLIG2 cells

The estimated mean OLIG2 cells/mm^2^ for treatment groups are shown in Figure 4, with group comparisons in Table 5. Over all brain regions, OLIG2 cells/mm^2^ were 30.8 cells/mm^2^ in HT, 29.1 cells/mm^2^ in HT+IV huMSC and 40.9 cell/mm^2^ in HT+IN huMSC groups. With regard to regional differences, there were higher OLIG2 counts in the internal capsule in the HT+IN huMSC group (99.5 cells/mm^2^) versus HT (74.6 cells/mm^2^, *P*=0.013) and HT+IV huMSC (78.9 cells/mm^2^, *P*=0.037) groups. There were higher OLIG2 counts in the hippocampus in the HT+IN huMSC group (45.8 cells/mm^2^) versus HT (18.6 cells/mm^2^, *P* < 0.011) and HT+IV huMSC (21.3 cells/mm^2^, *P*=0.018) groups. In the periventricular white matter, there were more OLIG2 cells in the HT+IN huMSC group (78.6 cells/mm^2^) compared with the IV huMSC group (54.4 cells/mm^2^, *P*=0.018).

**Table 5:** Oligodendrocyte cell counts/mm^3^.

Brain regions	HT	HT+IV huMSCs	HT+IN huMSCs	*P* values for differences in means	
cCTX	14.7 (6.8)	12.1 (6.8)	18.3 (6.8)	HT vs HT+IV huMSCs	0.852
				HT vs HT+IN huMSCs	0.759
				HT+IV huMSCs vs HT+IN huMSCs	0.517
sCTX	18.2 (6.8)	16.4 (6.8)	21.1 (6.8)	HT vs HT+IV huMSCs	0.852
				HT vs HT+IN huMSCs	0.759
				HT+IV huMSCs vs HT+IN huMSCs	0.622
Hippocampus	18.6 (7.3)	21.3 (6.8)	45.8 (7.3)	HT vs HT+IV huMSCs	0.788
				**HT vs** **HT+IN huMSCs**	**0.011**
				**HT+IV huMSCs vs HT+IN huMSCs**	**0.018**
pvWM	65.2 (6.8)	54.4 (6.8)	78.6 (6.8)	HT vs HT+IV huMSCs	0.8263
				HT vs HT+IN huMSCs	0.169
				**HT+IV huMSCs vs HT+IN huMSCs**	**0.015**
IC	74.6 (6.8)	78.9 (6.8)	99.5 (6.8)	HT vs HT+IV huMSCs	0.655
				**HT vs** **HT+IN huMSCs**	**0.013**
				**HT+IV huMSCs vs HT+IN huMSCs**	**0.037**
Caudate	13.7 (6.8)	13.8 (6.8)	18 (6.8)	HT vs HT+IV huMSCs	0.992
				HT vs HT+IN huMSCs	0.659
				HT+IV huMSCs vs HT+IN huMSCs	0.667
Putamen	14.9 (6.8)	13.7 (6.8)	18.4 (6.8)	HT vs HT+IV huMSCs	0.905
				HT vs HT+IN huMSCs	0.712
				HT+ IV huMSCs vs HT+IN huMSCs	0.626
Thalamus	26.7 (6.8)	22.5 (6.8)	27.4 (6.8)	HT vs HT+IV huMSCs	0.663
				HT vs HT+IN huMSCs	0.947
				HT+IV huMSCs vs HT+IN huMSCs	0.615
Overall	30.8 (4.9)	29.1 (4.9)	40.9 (4.9)	HT vs HT+IV huMSCs	0.811
				HT vs HT+IN huMSCs	0.173
				HT+IV huMSCs vs HT+IN huMSCs	0.116

cCTX, cingulate cortex; IC, internal capsule; pvWM, periventricular white matter; sCTX, sensorimotor cortex; SEM, standard error of the mean. Bold values denote statistical significance (P < 0.05)

#### GFAP, CC3, Iba1

**Fig. 5:**
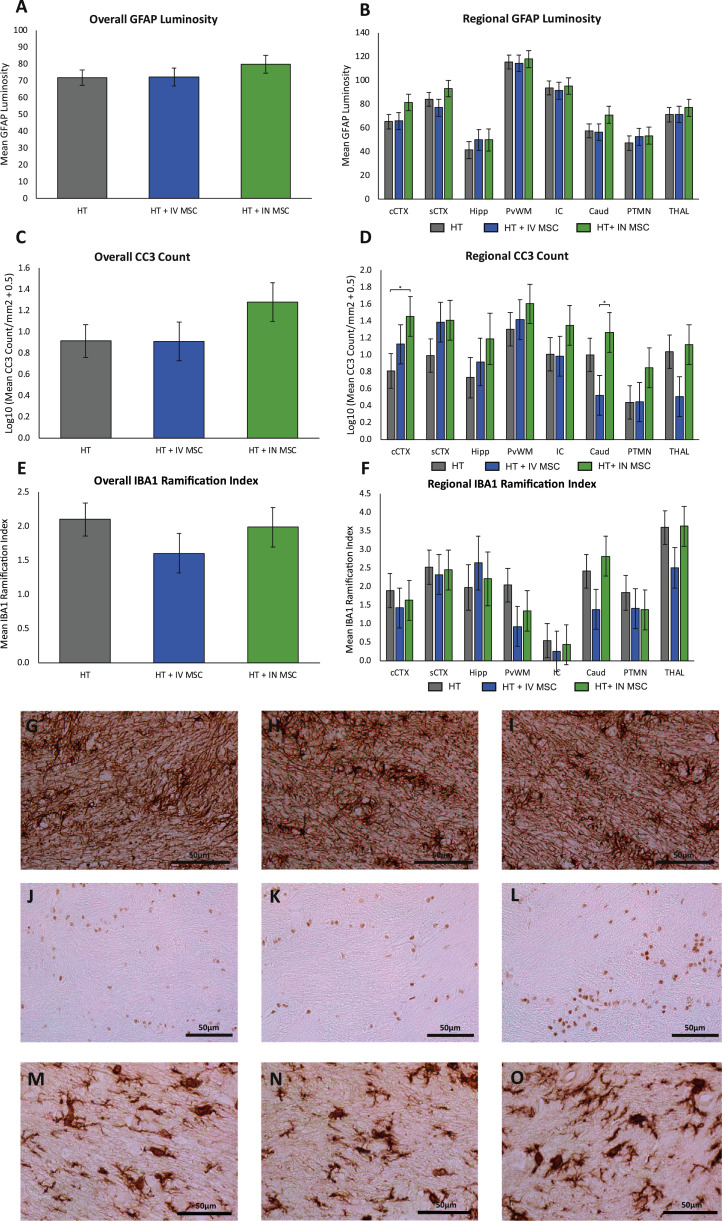
**Immunohistochemistry results for GFAP, CC3 and IBA-1.**GFAP luminosity is shown from all brain regions in (A) and in eight individual brain regions in (B). There was no difference in overall or regional GFAP luminosity. Representative GFAP photomicrographs from the IC of HT (G), HT+IV huMSC (H) and HT+IN huMSC (I) groups are shown. Mean CC3 cells/mm^2^ are shown from all brain regions in (C) and from eight individual brain regions in (D). There was no overall difference in CC3 across groups. Localized differences, with increased CC3, in the HT+IN huMSC group (n=5) compared with the HT group (n=7) were seen in cCTX (*P*=0.043) and caudate (*P*=0.031). Representative CC3 photomicrographs from the IC of HT (J), HT+IV huMSC (K) and HT+IN huMSC (L) groups are shown. Iba1 ramification index is shown from all regions in (E) and from eight individual brain regions in (F). There was no difference in overall or regional Iba1 ramification index. Representative Iba1 photomicrographs from the IC of HT (M), HT+IV huMSC (N) and HT+IN huMSC (O) groups are shown. *P < 0.05. Caud, caudate; cCTX, cingulate cortex; Hip, hippocampus; IC, internal capsule; PTMN, putamen; pvWM, periventricular white matter; sCTX, sensorimotor cortex; THAL, thalamus. (Color version of figure is available online).

There was no difference in overall or regional GFAP luminosity measuring astrogliosis (Figure 5). There was no overall difference in CC3 across all groups (Figure 5). Localized differences with increased CC3 in the HT+IN huMSC group compared with the HT group were seen in cingulate cortex (*P*=0.043) and caudate (*P*=0.031). There were no differences overall or regionally between groups for Iba1.

### Visualization of PKH huMSCs in the brain

**Fig. 6:**
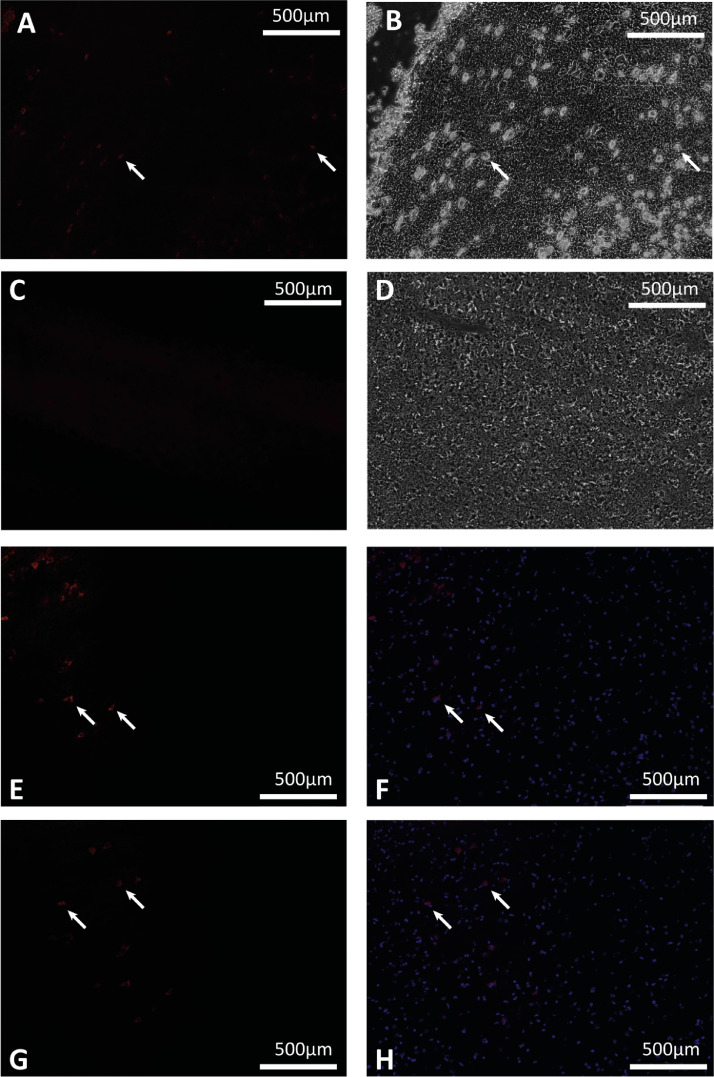
Representative photomicrographs of animals (n=2) treated with PKH26-labeled huMSCs, demonstrating the migration of IN huMSCs into the brain. (A) Photomicrograph of PKH26 fluorescence (arrows) and (B) corresponding brightfield photomicrograph with huMSC morphology (arrows). Photomicrograph of area with (C) no PKH26 fluorescence and (D) no corresponding huMSC morphology. (EH) Photomicrographs taken from each of the animals, showing PKH26 fluorescence (E,G) and corresponding PKH26/DAPI overlay (F,H), indicated by white arrows. (Color version of figure is available online).

MSC morphology was observed on coronal sections at R1 (10 m anterior to hippocampus) and R2 (a further 5 m anterior) under 10 magnification, and immunofluorescence confirmed the presence of PKH26-labeled huMSCs with DAPI nuclear co-stain in cryosections beginning 12 h after administration (Figure 6).

## Discussion

Compared with HT, the standard therapy for babies with NE, there was a more rapid aEEG recovery beginning 25 h after HI and improved brain energy metabolism (^31^P MRS PCr/Pi) on day 2 in the group given IN huMSCs at 24 h and 48 h with HT, although no difference was seen in the Lac/NAA measures on ^1^H MRS. Increased OLIG2 counts in hippocampus, internal capsule and periventricular WM and reduced TUNEL-positive cells in the internal capsule were seen with HT+IN huMSCs compared with HT. The authors detected PKH-labeled huMSCs in the brain after HI 12 h after IN administration. There was no effect of HT+IV huMSCs compared with HT alone. This 72-h study reports a modest augmentation of hypothermic neuroprotection with IN delivery of huMSCs in a large animal model of HI. Longer-term studies may be needed to detect the full therapeutic potential of huMSCs. Therapy such as IN huMSCs that can sense or adapt to brain injury would be a paradigm shift for the devastating effects of NE.

Administration of IV and IN stem cells was safe and did not lead to any acute physiological changes over the 72 h. There was no difference in requirements for saline boluses or inotropes to maintain MABP within normal limits. After IV infusion, MSCs are known to distribute to the lungs and, in susceptible patients, may be associated with transient increases in pulmonary pressure, leading to pulmonary edema [24]; the authors saw no change in the ventilation parameters with HT+IV huMSCs. Other known side effects of IV huMSCs, such as fever, cardiac dysfunction, arrhythmias, renal dysfunction and infection, were not associated with IV huMSCS in this 72-h study.

In this study, the authors used huMSCs, which have superior anti-inflammatory effects compared with adult MSCs [8]. MSCs have the potential to treat adult diseases such as stroke [25]. A meta-analysis of 46 pre-clinical studies of MSCs for ischemic stroke showed improved outcomes (behavioral and histologic), with large effect sizes [26]; positive results were seen across species, different routes of administration, dose and timing of administration. Improvements in quality of life and functional benefit have been seen in Phase 1 and 2 clinical studies [27], although challenges around comorbidities, age and stroke subtype are significant. MSCs have the potential for both early and late treatment in stroke, thus reducing injury and promoting repair. The total dose of huMSCs administered in the authors study (IV and IN) was 60נ10^6^ cells over 48 h after HI (~30נ10^6^ cells /kg), which is comparable to animal models [9] and previously published studies of MSCs for neonatal and pediatric diseases [7].

In the authors piglet model of perinatal HI, huMSCs were given at 24 h (after 12 h of HT and rewarming) and 48 h, thus targeting amelioration of HI injury rather than longer-term regeneration and repair. MSCs do not survive long-term or replace damaged tissues themselves, but rather auto-tune their response according to the brain's milieu [10]. MSCs are responsive to the needs of the brain's environment via cell-to-cell communication through paracrine secretion of growth factors and cytokines to regulate damage and repair. MSCs are specifically attracted to sites of injury and inflammation. After HI, tissues release inflammatory cytokines, and MSCs express cytokine receptors, homing to the sites of injury [28]. MSCs act through four main mechanisms: induction of cell proliferation, immunomodulation, angiogenesis and secretion of neurotrophic factors/reduction of cell death. Cell proliferation occurs by an upregulation of genes involved in neuroregeneration and angiogenesis as well as increased cell proliferation in the subventricular zone by mobilizing endogenous neuronal precursors [29]. In the P9 mouse model, IN MSCs were seen to promote the formation of a neurogenic niche in the subventricular zone, inducing and supporting the shift to a neurogenesis supportive environment [30]. Immunomodulation by MSCs reduces damaging inflammation that impedes repair, changing macrophage profiles to become less injurious [31,32], although laboratory cell-based immunosuppression assays do not always correlate with patient outcomes in, for example, graft-versus-host disease [33]. In the authors study, the relatively short duration may have missed a potentially important immunomodulatory effect of HT+IN huMSCs compared with HT, as no difference was seen in Iba1 staining. Despite entrapment in the lungs, IV MSCs have been shown to have systemic immunomodulatory effects on distant organs, including the brain [34], although this was not seen in the authors study. Secretion of neurotrophic factors, such as brain-derived neurotrophic factor, enhances survival of and reduces apoptosis in neurons, enhances differentiation of endogenous neural progenitors, activates astrocytes and reduces scar formation and promotes remodeling of neurons and glial and vascular cells [9]. In the authors 72-h study, we saw increased survival of oligodendrocytes in white matter and reduced TUNEL-positive cells in the internal capsule but no effect on astrogliosis with HT+IN huMSCs.

Absence of benefit from IV-huMSCs in the authors study concurs with the reported unfavorable biodistribution of IV stem cells reported in pre-clinical studies, with only ~2.5% of cells passing through the pulmonary vessels [35,36]. However, several stroke studies have shown benefit of the IV route [26], probably through systemic immunomodulation. A significant benefit of IN delivery of huMSCs is the ability to bypass the blood-brain barrier and directly enter the central nervous system [37]. IN MSCs have been shown to migrate from the cribriform plate along the olfactory neural pathway or trigeminal and perivascular routes into the brain and cerebrospinal fluid [38]. The rapid migration of stem cells [39] or drugs [40] has been observed in adult and neonatal [30] pre-clinical models of brain protection. The time course of migration has been observed following HI in the neonatal 9-day mouse model using PKH-labeled cells and immunofluorescence; MSCs were seen to reach the lesion site in the brain as early as 2 h after administration, with a peak at 12 h [30]. In two piglets in the current study, the authors euthanized at 12 h after PKH huMSC administration and were able to visualize cell immunofluorescence, demonstrating a similar time course of cell migration. Such PKH labeling may have, however, diminished the huMSC migration compared with non-labeled huMSCs [41].

Several pre-clinical rodent studies support the potential of stem cells to treat NE; these cells have been administered intracranially [42], intraperitoneally [43] and intranasally [44,45]. Administration of MSCs at 3 and 10 days after HI in the P9 mouse model indicated possible additive effects from repeat doses, with the early dose primarily influencing cell proliferation and the second dose acting via different pathways involved with injury response and brain development [42]. Augmentation of HT with transplanted intraventricular MSCs has been observed in P7 rats following HI, with cooling and cell transplantation starting at 6 h after HI [46]. Augmented protection was based on reduced infarct volume, TUNEL-positive cells, cerebrospinal fluid cytokine levels and improved behavioral tests [46]. The same group showed that even if MSC transplantation was delayed to 2 days after HI, robust synergy between cooling and MSCs persisted [47]. However, a study in P9 mice with 4-h HT after HI and MSCs administered intranasally at P12 showed exacerbation of brain injury, with higher pro-inflammatory cytokine levels [48]. These unexpected data suggest that the combination of HT with MSCs in this model shifted the brain's milieu to a pro-inflammatory environment, with an alteration of growth factor balance.This study emphasizes the importance of careful assessment of the safety of the combination of MSCs with HT before clinical trials in babies with NE.

In this current study in male piglets, the authors saw increases in surviving oligodendrocytes in the periventricular white matter and internal capsule in the HT+IN huMSC group compared with HT, suggesting that, at 72 h, the main target of IN huMSCs, over and above HT, is white matter. Improved oligodendrocyte survival was also seen in the hippocampus with HT+IN huMSCs compared with HT. A P9 mouse model with MSC treatment at 3 days after injury also demonstrated oligodendrocyte regeneration as well as functional improvement and reduced lesion size [42]. The authors of the current study saw reductions in TUNEL-positive cells in the internal capsule with HT+IN huMSCs compared with HT. These data concur with previous studies that have consistently shown MSCs reduce apoptosis and increase proliferation of several cell types [29]. The authors saw no difference in microglial activation overall or regionally; given the key role of MSCs in immunomodulation [8], this was surprising. As evolving damage from inflammation can last weeks to months, an effect on neuroinflammation from huMSCs may become more apparent in longer-term studies [31]. The authors saw no difference in GFAP luminosity; given the importance that astrocytes play in mediating central nervous system plasticity and neurological recovery after HI injury, these differences may be detected in future studies of longer duration [49]. The authors saw some localized increases in CC3 in the cingulate cortex in HT+IN huMSCs compared with HT, but as in previous studies in the male piglet model, at 72 h [50] CC3 is a poor marker of cell death/apoptosis and is likely to reflect caspase's non-apoptotic functions, such as promoting microglial and lymphocyte function, cell differentiation and autophagy [51,52]. The authors have previously observed discrepancies between TUNEL-positive cell death and CC3 [50]. The use of male piglets may partly explain these data, as cell death is dimorphic, with apoptosis occurring via caspase-independent pathways in males [53,54].

There are limitations to the authors study. The inclusion of only male piglets was to minimize variability; larger studies with both male and female piglets will be important in future investigations. For rodents and piglets, the total area of the olfactory epithelium occupies ~50% of the nasal mucosal area [55], whereas the olfactory region occupies only 12% of the nasal cavity surface area in human beings. This may influence the ability of MSCs to migrate to the brain in babies with NE; however, IN therapy in humans is already successfully performed for drugs and peptides [37], and IN MSC migration has been observed in a neonatal primate model of HI [9]. The authors piglets were anesthetized throughout the study; it is unclear if anesthesia influences IN delivery. The authors did not use hyaluronidase to disrupt the nasal epithelium and aid MSC penetration as in previous rodent studies [30,45]; this may have reduced huMSC brain penetration. Higher doses of systemically administered MSCs may be needed compared with local administration to allow for potential loss of MSCs in peripheral organs [9]. The authors used the same dose in both IV and IN administration and so did not allow for this possible need for increased dose of huMSCs with systemic administration. Indeed, in one P7 rat study with IV human umbilical cord blood cells given 24 h after injury, there was a clear dose-dependent response for tissue repair [56]. Cells for the IV huMSC group were cryopreserved in DMSO, whereas cells for IN huMSCs were in non-DMSO solution to avoid local irritation. Although the MSCs were from a single umbilical cord, the authors data show consistency between cords of individual donors and retention of characteristics when pooled and expanded to >40 population doublings.

The main limitation of this study was its relatively short duration (72 h) compared with rodent studies, and so the authors were able to study potential amelioration of injury but not regeneration, repair and immunomodulatory effect of huMSCs. Nevertheless, the authors were able to see evidence of early amelioration of injury and some evidence of oligodendrocyte proliferation/survival with HT+IN huMSCs. Importantly, the authors did not see exacerbation of injury, as in the mouse model of HT and MSCs [48]; however, pro-inflammatory cytokine profiles were not studied here. The authors observed improvement in whole brain PCr/Pi but not ^1^H MRS Lac/NAA sampled from both white and gray matter. This absence of a visible effect of HT+IN huMSCs on Lac/NAA in the WM voxel was surprising and may be related to the smaller voxel sizes and sampling of less white matter tissue with ^1^H MRS compared with ^31^P MRS. The PKH-labeled huMSCs were administered at 1 h after HI and not at 24 h, as in the main study, and this could have affected the rate or ease of infiltration of cells into the brain.

The main strengths of this study were its standardized HI insult, known timing of injury and clinically relevant outcome measures [14]. We wished to assess the effect of MSC therapy started after the cooling and rewarming period; we therefore shortened the period of cooling from our usual protocol of 24h [57] to 12h which has also been seen to be effective in this model [58].

IN-huMSC therapy is a potential paradigm shift in the treatment of the devastating effects of NE. Over the last decade, there has been considerable focus on adjunct therapies for HT in babies with moderate to severe NE [5]. Phase 3 clinical trials are ongoing with erythropoietin (NCT02811263) and allopurinol (NCT03162653) as an adjunct with HT. A phase 2 study in 2015 assessed the safety and efficacy of two IV infusions of autologous red blood cell-reduced nucleated umbilical cord blood cells compared with placebo in 36 babies with NE undergoing HT (NCT02612155). Other agents, such as melatonin [18], xenon [59], exendin-4 [60] and azithromycin [61], are in the pre-clinical phases. These therapies require an IV line, cold storage or a ventilator. Unlike huMSCs, these therapies are static and may not be responsive to different injury severities, inflammatory milieu or sex. The IN route of huMSCs has the potential to tailor to the specific milieu of the injured brain, and as the cells are allogeneic and off the shelf, urgent laboratory processing, as with autologous cells, is not required.

### Conclusions

Administration of two doses of 30 million IN huMSCs at 24 h and 48 h after HI (total dose 30נ10^6^ cells) was safe and modestly augmented brain protection in a piglet model of perinatal asphyxia undergoing cooling. Protection with IN HT+huMSCs compared with HT alone was based on more rapid recovery of aEEG and improved brain energy metabolism on ^31^P MRS but not ^1^H MRS imaging biomarkers. White matter protection was indicated by improved oligodendrocyte survival and localized reduced TUNEL-positive cells. PKH-labeled huMSCs were detectable in the brain 12 h after HI. There was no evidence of benefit with the same dose of HT+huMSCs given intravenously compared with HT. IN huMSCs are a potentially powerful therapeutic option that holds promise as a paradigm shift in the treatment of babies with moderate to severe NE.

## Appendix Supplementary materials

Image, application 1
