# Supplementary material for: Human umbilical cord mesenchymal stromal cells as an adjunct therapy with therapeutic hypothermia in a piglet model of perinatal asphyxia
Source: Cytotherapy. 2021 Jun;23(6):521–35. doi: 10.1016/j.jcyt.2020.10.005 (PMC8139415; doi:10.1016/j.jcyt.2020.10.005)
Supplement: Supplementary file 1 [file mmc1.docx]

**Assessment of MSC functionality**

Immunomodulatries capacities of MSCs were assessed by T cell supression assay using PBMC. The figure below shows the results obtained by flow cytometry using CFSE with co-culture of two MSC donors and activated-PBMC for 3 batches. As shown in the table below, the inhibition of proliferation of lymphocytes cells are at least of 30% when MSC are added to PBMC.

**Donor 1**


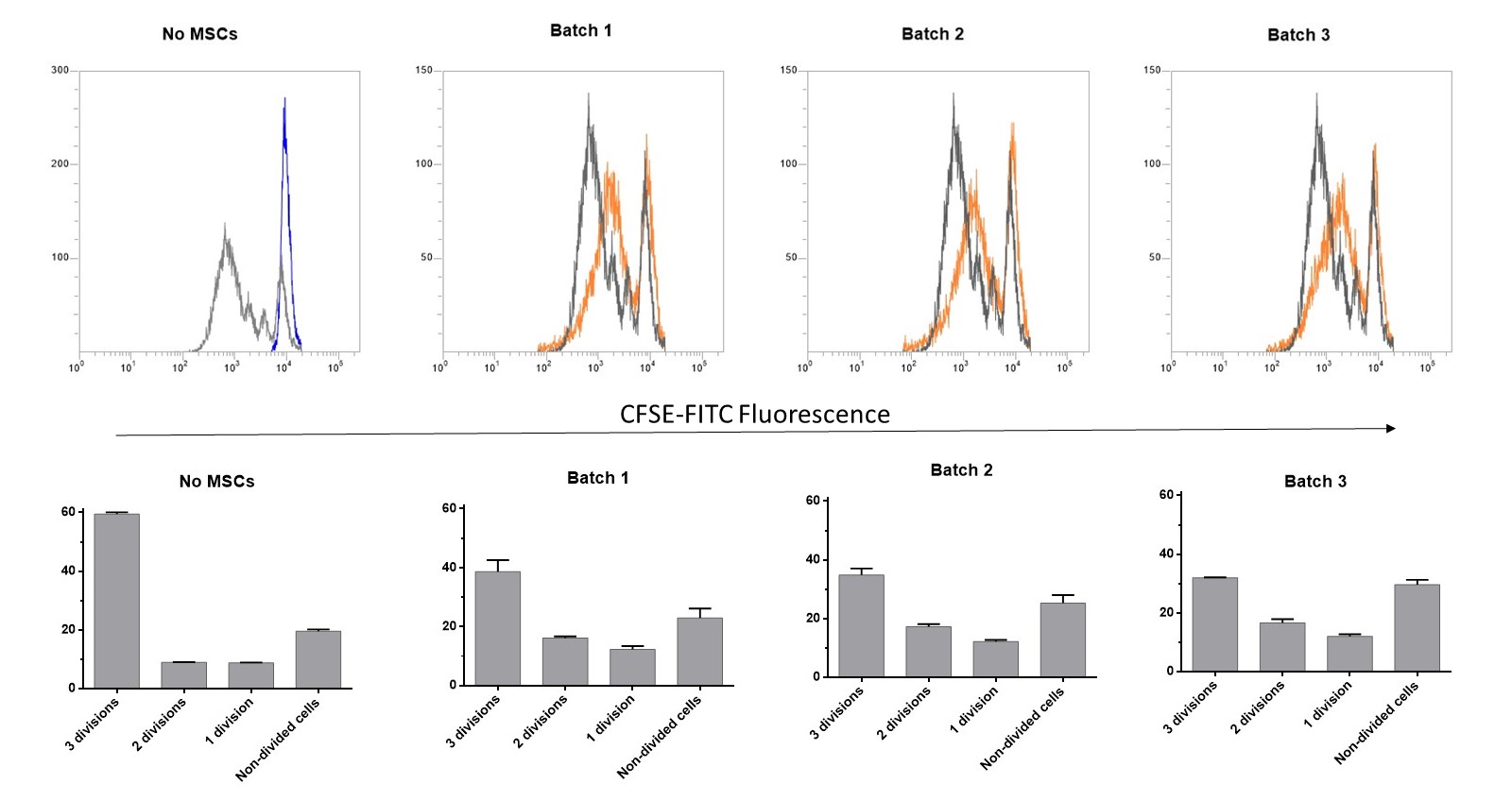


(H)

(G)

(F)

(E)

(D)

(A)

(C)

(B)

**Donor 2**


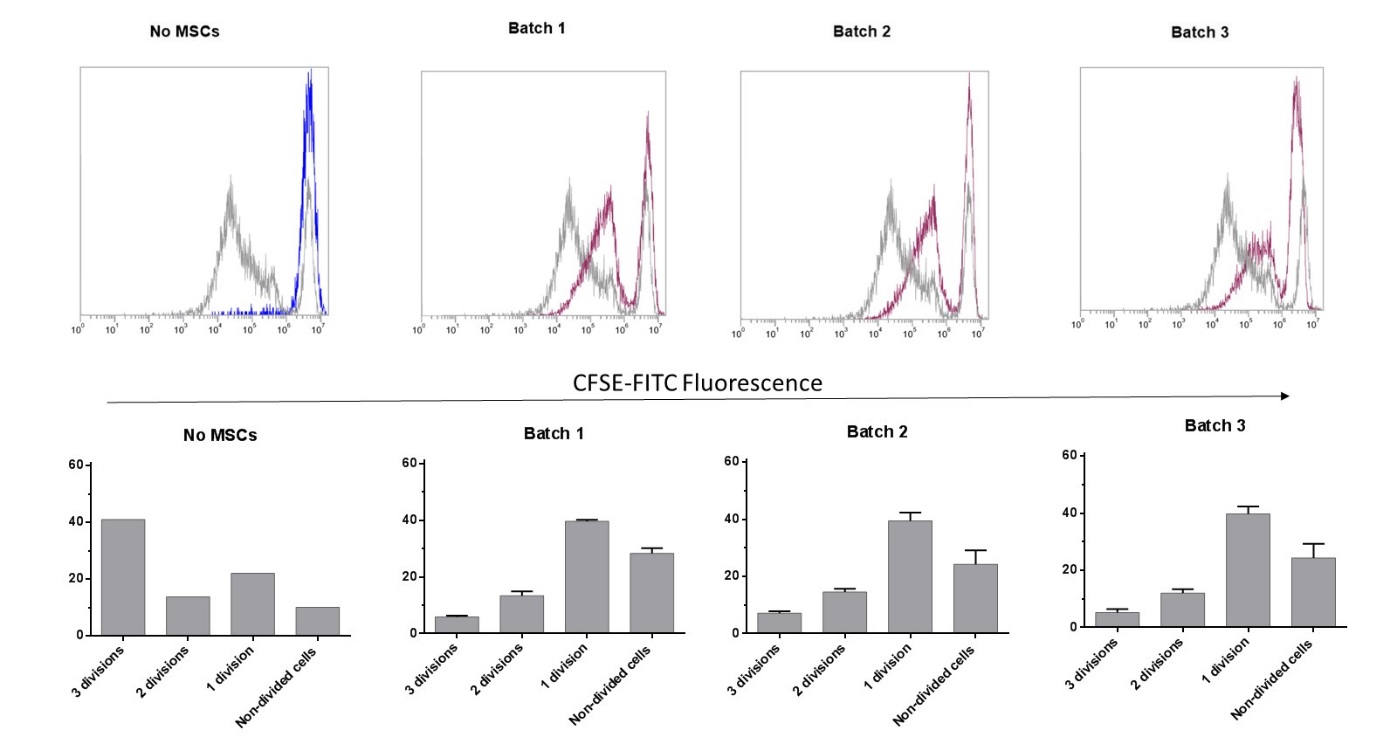


(M)

(P)

(O)

(N)

(I)

(L)

(K)

(J)

**Figure:** Effect of MSCs upon proliferation distributions of PBMCs.

Shown are CFSE histograms for (A, I) PBMCs only (blue), and activated PBMCs in which responder cells have undergone 3 cell divisions (grey). Histograms for cell cultures in which 1:3 MSC to PBMCs were added are shown in B-P (orange), overlayed against the plot of PBMCs only (grey) for batch 1, 2 and 3 for two different umbilical cord tissue derived-MSC donors. The mean (±SD) percentage distribution of PBMCs in each cell division is shown in bar charts (E-H, M-P); cell division is noted to reduce with MSC addition (F-H,N-P), corresponding to the proposed immunomodulatory function of MSCs.

**Table**: CFSE-derived metrics to characterise MSC suppression of PBMC proliferation.

**Donor 1**

|  | Mean percentage change in the 3^rd^ cell division fraction following MSC addition | Mean percentage change in the non-dividing PBMC fraction following MSC addition |
| --- | --- | --- |
| Batch 1 | -34.99% | 17.16% |
| Batch 2 | -41.42% | 28.79% |
| Batch 3 | -46.25% | 50.68% |

Donor 2

|  | Mean percentage change in the 3^rd^ cell division fraction following MSC addition | Mean percentage change in the non-dividing PBMC fraction following MSC addition |
| --- | --- | --- |
| Batch 1 | -85.54% | 181.43% |
| Batch 2 | -82.53% | 141.21% |
| Batch 3 | -87.34% | 142.11% |
